# Supplementary figures and images for: Feeding ecology and trophic interactions of the narrow-barred Spanish Mackerel (Scomberomorus commerson) in the Central Taiwan Strait
Source: PeerJ. 2025 Nov 14;13:e20350. doi: 10.7717/peerj.20350 (PMC12622235; doi:10.7717/peerj.20350)

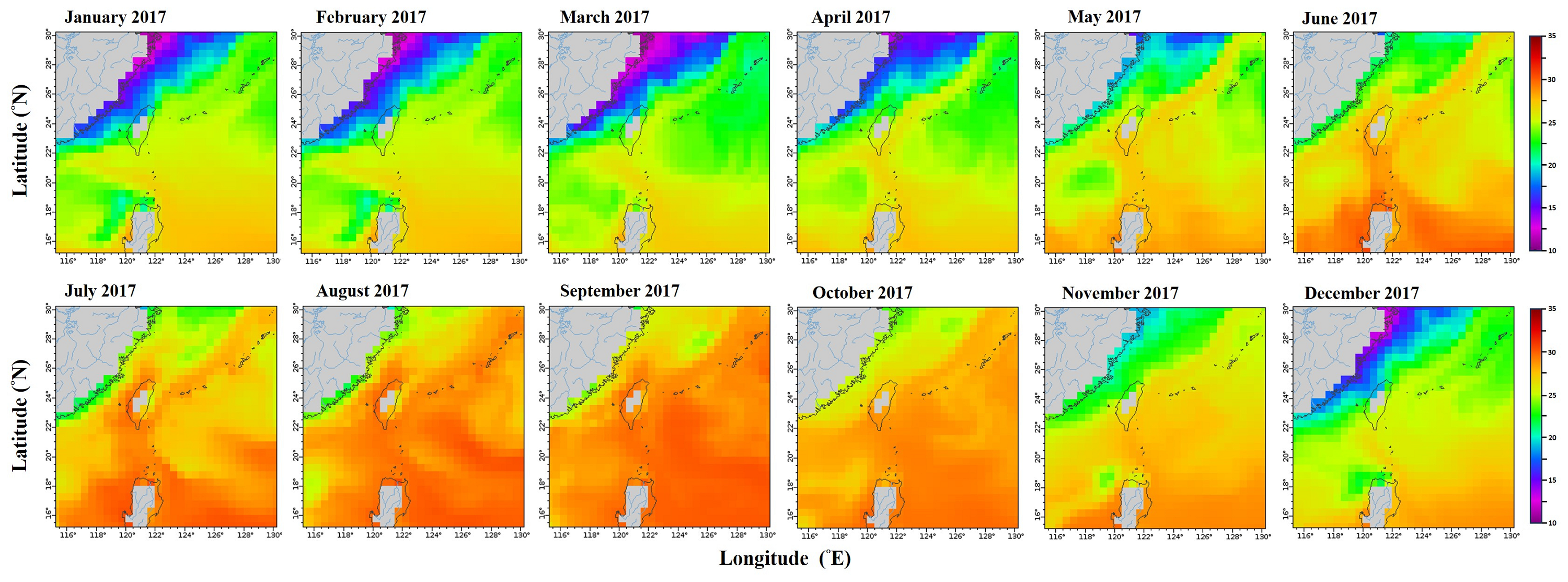

Supplement: Supplemental Information 1 — Data retrieved from the ERDDAP server hosted by the Asia-Pacific Data-Research Center (APDRC), University of Hawaii. Map Source: http://apdrc.soest.hawaii.edu/erddap/griddap/hawaii_soest_f88c_2508_4a21.graph). [file peerj-13-20350-s001.png]

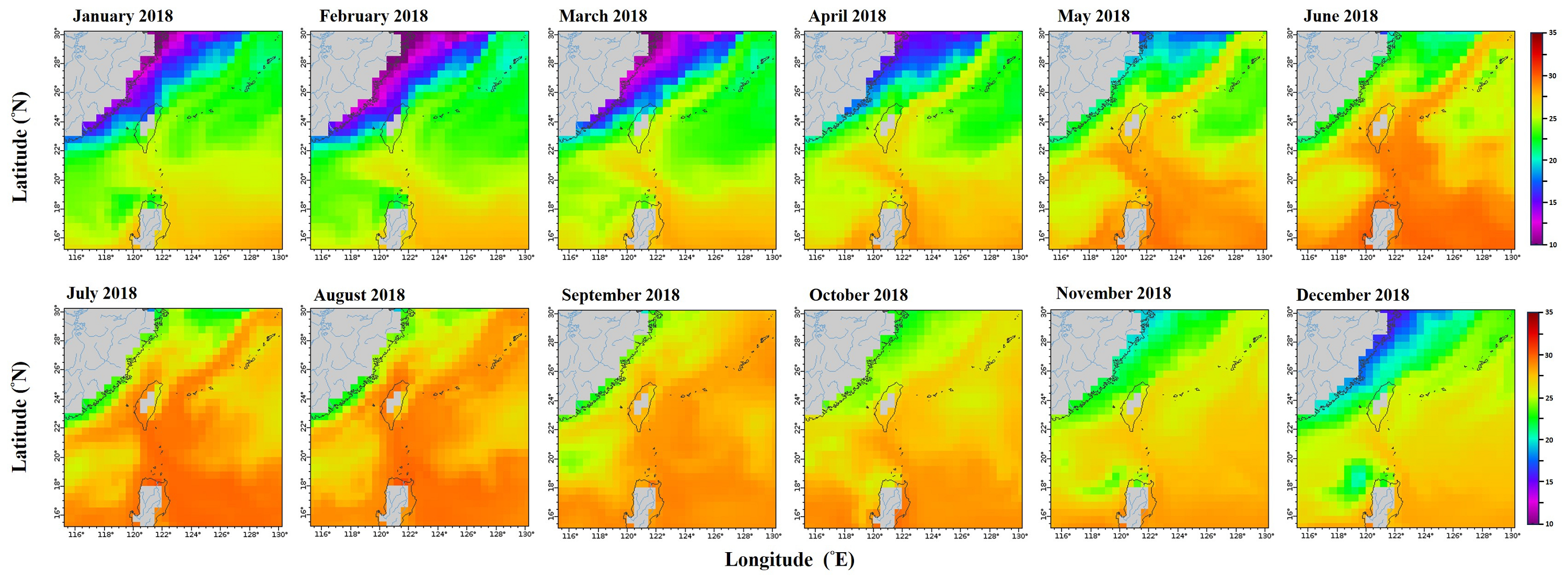

Supplement: Supplemental Information 2 — Data retrieved from the ERDDAP server hosted by the Asia-Pacific Data-Research Center (APDRC), University of Hawaii. Map Source: http://apdrc.soest.hawaii.edu/erddap/griddap/hawaii_soest_f88c_2508_4a21.graph). [file peerj-13-20350-s002.png]

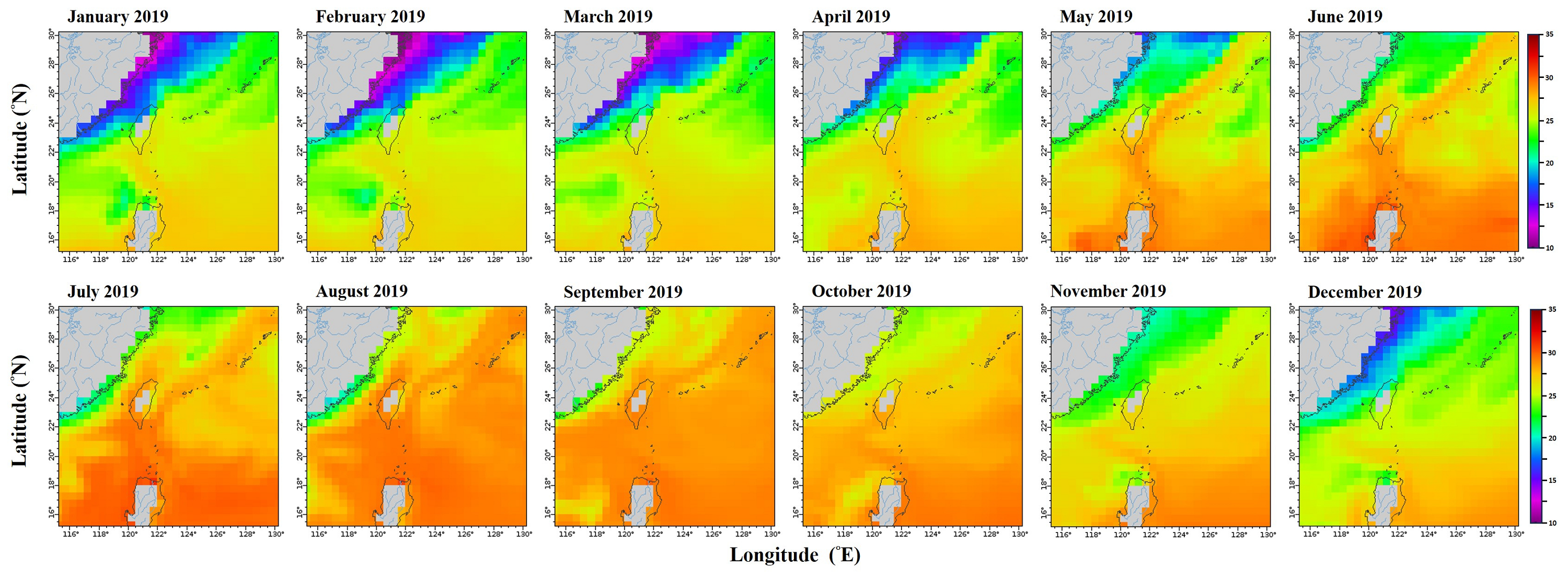

Supplement: Supplemental Information 3 — Data retrieved from the ERDDAP server hosted by the Asia-Pacific Data-Research Center (APDRC), University of Hawaii. Map Source: http://apdrc.soest.hawaii.edu/erddap/griddap/hawaii_soest_f88c_2508_4a21.graph). [file peerj-13-20350-s003.png]

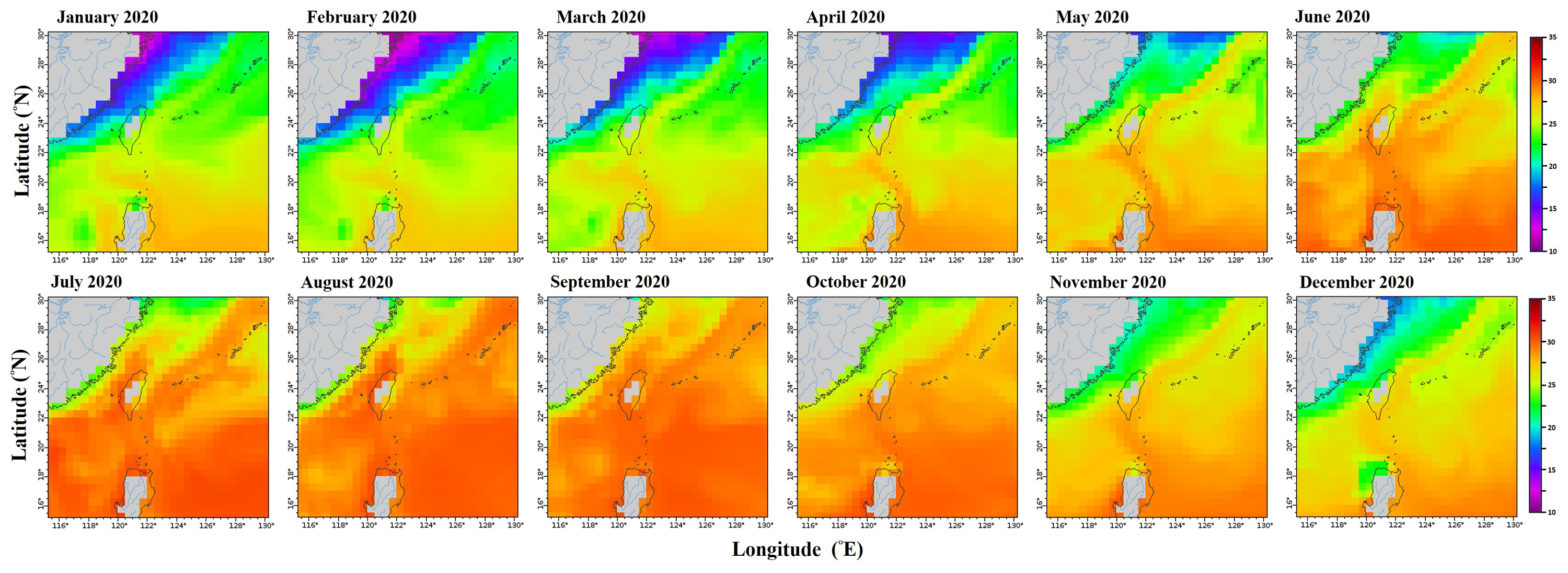

Supplement: Supplemental Information 4 — Data retrieved from the ERDDAP server hosted by the Asia-Pacific Data-Research Center (APDRC), University of Hawaii. Map Source: http://apdrc.soest.hawaii.edu/erddap/griddap/hawaii_soest_f88c_2508_4a21.graph). [file peerj-13-20350-s004.png]

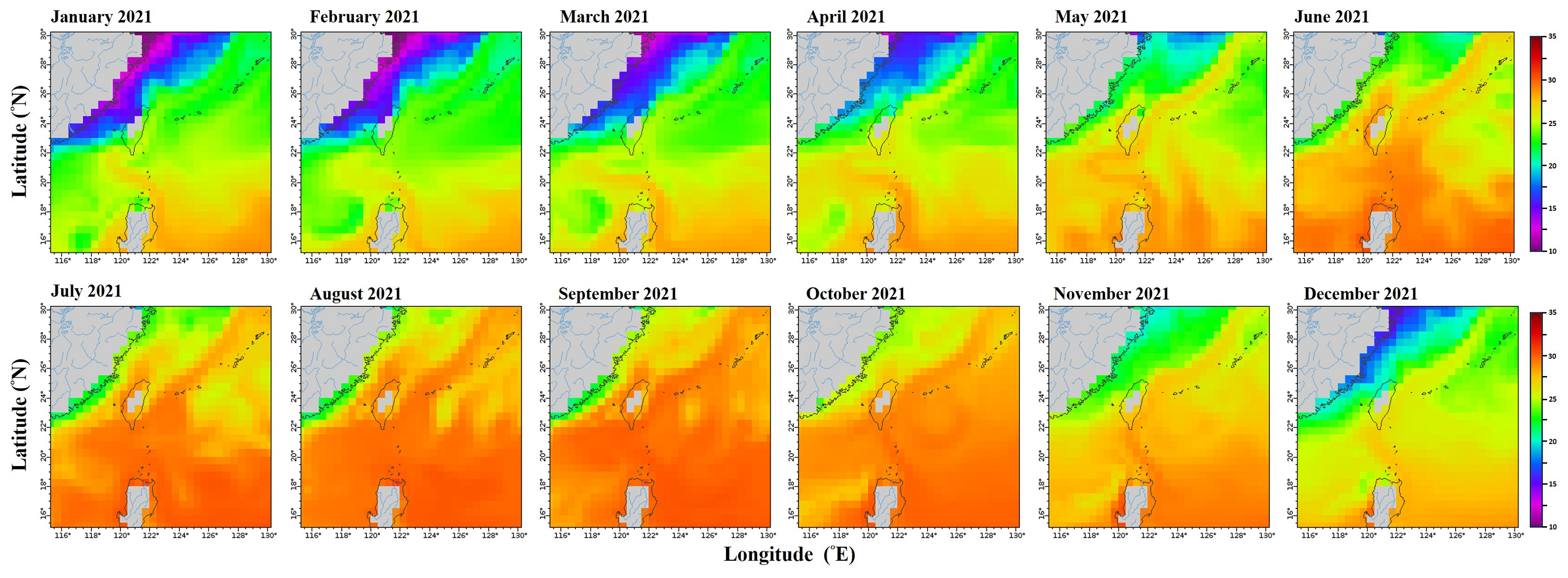

Supplement: Supplemental Information 5 — Data retrieved from the ERDDAP server hosted by the Asia-Pacific Data-Research Center (APDRC), University of Hawaii. Map Source: http://apdrc.soest.hawaii.edu/erddap/griddap/hawaii_soest_f88c_2508_4a21.graph). [file peerj-13-20350-s005.png]

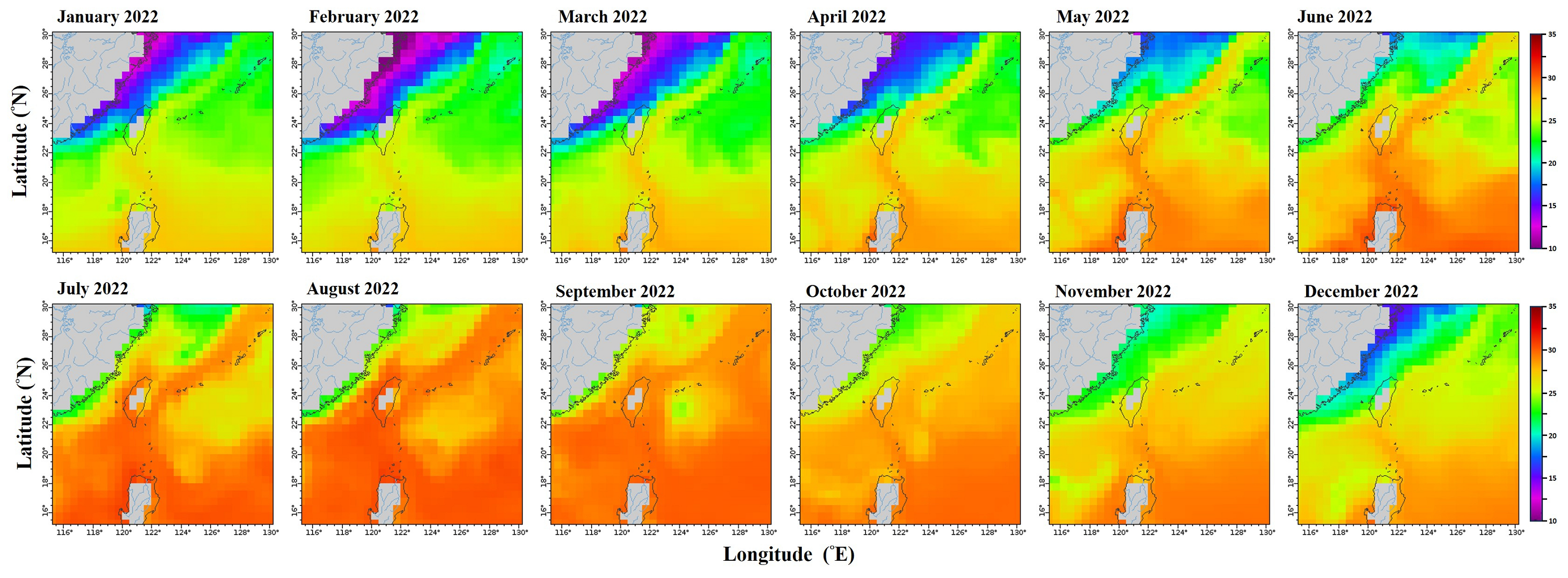

Supplement: Supplemental Information 6 — Data retrieved from the ERDDAP server hosted by the Asia-Pacific Data-Research Center (APDRC), University of Hawaii. Map Source: http://apdrc.soest.hawaii.edu/erddap/griddap/hawaii_soest_f88c_2508_4a21.graph). [file peerj-13-20350-s006.png]

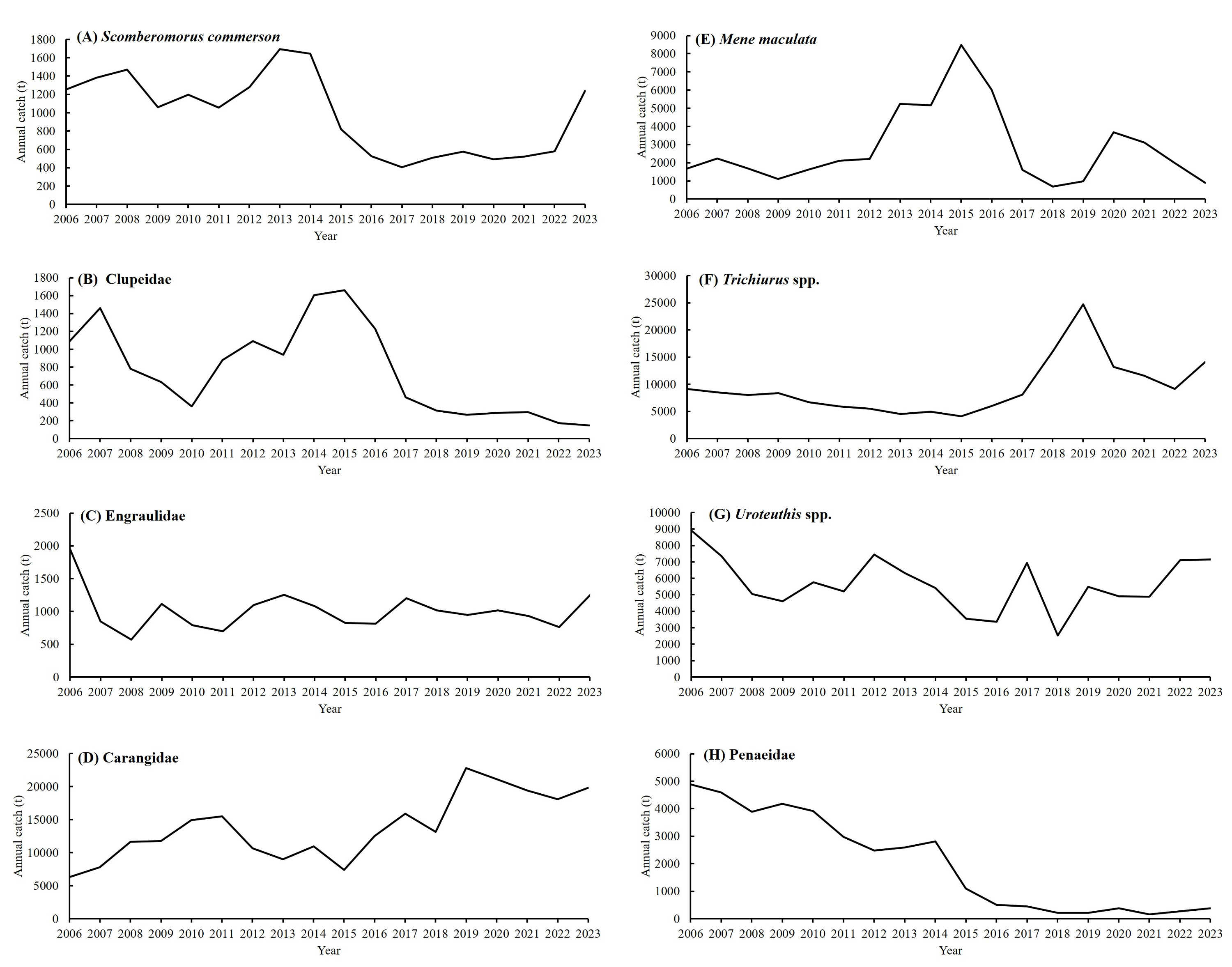

Supplement: Supplemental Information 7 — Data source: Taiwan (Fisheries Agency, 2025). [file peerj-13-20350-s007.png]
